# Dissociable roles of left and right temporoparietal junction in strategic competitive interaction

**DOI:** 10.1093/scan/nsz082

**Published:** 2019-10-31

**Authors:** Akitoshi Ogawa, Tatsuya Kameda

**Affiliations:** 1 Faculty of Medicine, Juntendo University, Tokyo 113-8421, Japan; 2 Brain Science Institute, Tamagawa University, Tokyo 194-8610; 3 Labratory for Symbolic Cognitive Development, RIKEN Center for Biosystems Dynamics Research, Saitama 351-0198, Japan; 4 Department of Social Psychology, The University of Tokyo, Tokyo 113-0033, Japan

**Keywords:** fMRI, temporoparietal junction, computational model, theory of mind, machine learning

## Abstract

Although many studies have shown that the temporoparietal junction (TPJ) is involved in inferring others’ beliefs, neural correlates of ‘second-order’ inferences (inferring another’s inference about one’s own belief) are still elusive. Here we report a functional magnetic resonance imaging experiment to examine the involvement of TPJ for second-order inferences. Participants played an economic game with three types of opponents: a human opponent outside the scanner, an artificial agent that followed a fixed probabilistic strategy according to a game-theoretic solution (FIX) and an artificial agent that adjusted its choices through a machine-learning algorithm (LRN). Participants’ choice behaviors against the human opponent and LRN were similar but remarkably different from those against FIX. The activation of the left TPJ (LTPJ) was correlated with choice behavior against the human opponent and LRN but not against FIX. The overall activity pattern of the LTPJ for the human opponent was also similar to that for LRN but not for FIX. In contrast, the right TPJ (RTPJ) showed higher activation for the human opponent than FIX and LRN. These results suggest that, while the RTPJ is associated with the perception of human agency, the LTPJ is involved in second-order inferences in strategic decision making.

## Introduction

Competitive situations in which one’s own benefit means the opponent’s loss (and vice versa) are common in many facets of our social lives, including resource allocation, rivalry for social status, games such as football or chess, public debate and so on. When such competitive situations are repeated, we must not only learn and predict the opponent’s behavior but also simultaneously be aware that the opponent also learns and predicts our behavior. This bilateral (‘higher-order’) inference about the opponent’s state of mind is a major ingredient in human strategic interactions.

A rational solution to such a competitive game is a mixed strategy that assigns a choice probability to each of the options according to the Nash equilibrium (Nash, 1950). However, it is known that humans often deviate from the equilibrium (Martin *et al.,* 2014; see also Sonsino, 1997). Rather than adopting the theoretically derived, fixed Nash strategy, people often seem motivated to explore the choice probabilities of the opponent for possible exploitation (McCabe *et al.,* 2001; Gallagher *et al.,* 2002; Sanfey *et al.,* 2003; Rilling *et al.,* 2004). However, such attempts at exploitation must take into account how our own choices may influence the opponent’s strategy (Hampton *et al.,* 2008; Hill *et al.,* 2017) and also reason about how the opponent may infer our current strategies (Coricelli and Nagel, 2009). In contrast, such bilateral, higher-order processes may not be invoked when people play against a computer opponent operating according to some fixed strategy. In other words, human choice behavior in a competitive situation can be expected to depend heavily on the nature of the opponent and how the opponent makes decisions.

Many neuroimaging studies have shown that the temporoparietal junction (TPJ) is associated with ‘theory of mind’—the ability to infer the agency and mind state of another (Saxe and Kanwisher, 2003; Saxe and Wexler, 2005; Saxe, 2006; Völlm *et al.,* 2006; Van Overwalle, 2009; Van Overwalle and Baetens, 2009; Schnell *et al.,* 2011; Van Overwalle, 2011; Takahashi *et al.,* 2014). The right TPJ (RTPJ) has been suggested to be involved in inferring others’ mental states in various social situations (e.g. moral judgment, Young and Saxe, 2009; distribution, Kameda *et al.,* 2016; risky decision making, Ogawa *et al.,* 2018). The left TPJ (LTPJ) is also important for representing and inferring another’s beliefs (Samson *et al.,* 2004; Biervoye *et al.,* 2016). Human imaging studies have suggested that the LTPJ is associated with attending to the gap in perspective between self and other (Perner *et al.,* 2006; Schurz *et al.,* 2013; Arora *et al.,* 2015). Although the functional roles of TPJ in inferring others’ beliefs have been progressively revealed, details remain elusive concerning the TPJ’s involvement in the higher-order recognition that others also infer one’s own beliefs during strategic decision making.

In this functional magnetic resonance imaging (fMRI) study, we investigated how the bilateral TPJs associated with ToM were involved in competitive decision making, in which correct inference of another’s inference about one’s own belief is essential. Participants played an economic game (the ‘asymmetric matching pennies game’: Martin *et al.,* 2014) in the MRI scanner against three types of opponents: a human opponent who played the game from outside the scanner and two artificial agents (FIX and LRN) whose choices followed computer algorithms (which was explicitly noted in participant instructions). Thus, neither FIX nor LRN involved human agency, and their choice algorithms were quite different from each other. FIX followed a fixed probabilistic mixed strategy according to a game-theoretic Nash equilibrium, without any inference of the participant’s strategy. On the other hand, LRN was programmed to predict the participant’s choice strategy and adjust its own choices using a machine-learning technique. As both the human opponent and LRN were expected to be responsive to the participant’s choices in a bilateral manner, we expected that the participant’s choice behaviors against the human opponent and against LRN would be similar to each other, but different from those used against FIX. Thus, we investigated how the neural activities of RTPJ and LTPJ were associated with both the perception of human agency and the higher-order recognition of the other agent’s inference about one’s own strategy in the competitive game.

## Methods

### Subjects

We scanned 30 right-handed student participants (14 females and 16 males; aged 18 to 22 years, mean = 19.3 years) at the University of Tokyo with no history of neurological or psychiatric illness. Thirty gender-matched, right-handed students (aged 18 to 22 years, mean = 19.4 years) also participated in this experiment as human opponents who played the game outside the scanner. The study was approved by the ethical committee of the Department of Social Psychology in the University of Tokyo. All participants gave written informed consent prior to the experiment.

### Task

The participants played the ‘asymmetric matching pennies game’ in the MRI scanner with three types of opponents: the human opponent (HUM) who played the game outside the scanner and the two artificial agents (FIX and LRN). FIX’s choices were determined stochastically according to the mixed Nash strategy, while LRN used a machine-learning algorithm to attempt to predict and exploit the participant’s choices. In the instructions, the human opponent was described as a student of the same sex at the same university, FIX as a computer program that would always follow a fixed, economically rational strategy and LRN as a computer program that would constantly learn to predict the participant’s choices through interaction.

**Fig. 1:**
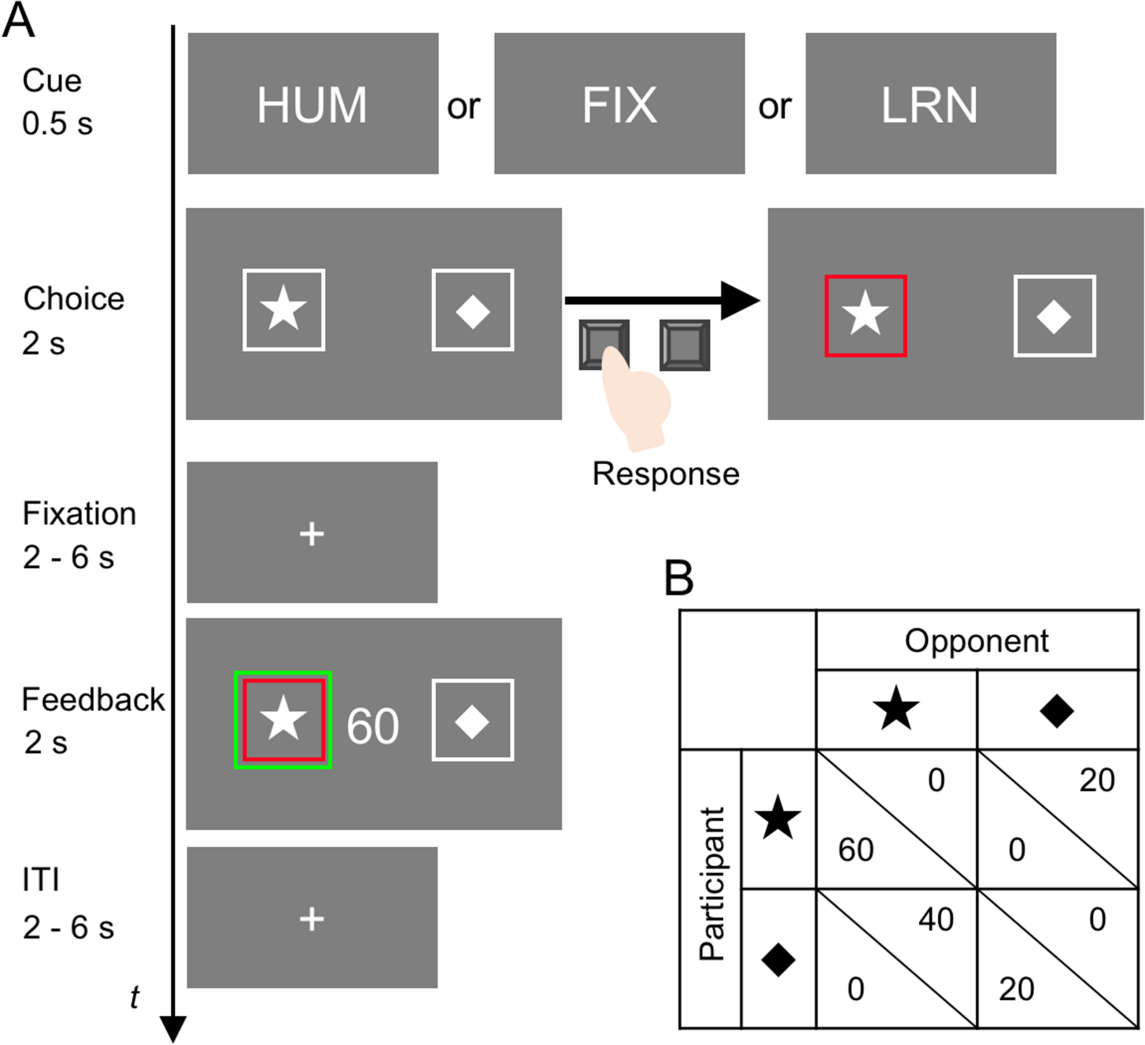
Task sequence (A) and payoff matrix (B). A. In each trial, a conditional cue was presented for 0.5 s. Two options were presented on the left and right sides of the display for 2 s. When the participant pressed a button, the frame of the chosen option was colored red. After a jittered fixation duration, the choice of the opponent was shown as a green frame along with the monetary outcome the participant obtained. ITI = inter-trial interval. B. The participant won the game when his/her choice matched with the opponent’s choice. The participant received 60 JPY by winning with the left choice (‘star’) and 20 JPY by winning with the right choice (‘diamond’) but received nothing for losing the game. The combination of left/right, star/diamond and outcome amounts was counterbalanced across participants.

At the beginning of each trial, a cue indicating the type of opponent (HUM, FIX or LRN) was presented for 0.5 s (Figure 1A). Then two choice options were presented on the left and right sides of the display. The participant was asked to choose either option within 2 s. Immediately after either button was pressed, the frame of the chosen option was colored red. After a jittered fixation duration (2, 4 or 6 s), the choice of the opponent was indicated by a green frame, along with the outcome amount for the participant displayed at the center. After a jittered inter-trial interval (ITI) (2, 4 or 6 s), the next trial began.

The participant won the game and received a monetary reward when their choice matched the opponent’s choice. As shown in Figure 1B, the participant received 60 JPY if both participant and opponent selected the left choice (‘star’ in this example) and 20 JPY if both selected the right choice (‘diamond’), while the opponent received nothing in either case. In contrast, when the two selections did not match, the participant received nothing, while the opponent received 40 JPY for ‘star’ or 20 JPY for ‘diamond’. Thus, the game payoff was asymmetric between the two players. We adopted this asymmetric payoff to make the game-theoretic probabilistic mixed strategy different from a random 50–50 choice; if the payoffs were symmetrical, it would be impossible to distinguish the game-theoretically rational strategy from a purely random choice (Camerer, 2003; Martin *et al.,* 2014). The combination of left/right sides, star/diamond shapes and outcome amounts was counterbalanced across participants. The participant’s understanding of the payoff matrix was confirmed by a series of quizzes prior to the experiment.

The choice behavior in this study is characterized by how the participants balance between choosing the option (‘star’ in the example) that could yield the higher outcome (high choice, hereafter) and the option (‘diamond’) with the lower outcome (low choice). As mentioned earlier, we expected that participants’ high choice rates in the HUM condition would be more similar to those in the LRN condition than in the FIX condition.

Each of four fMRI runs included 3 blocks, with 12 consecutive trials in each block. During each block, the participant played the game against the same opponent. The order of the opponent blocks was counterbalanced across participants. The participants performed 144 trials in total (i.e. 48 trials for each of the three opponents). The participants received the accumulated monetary outcomes in the experiment as a bonus in addition to a fixed compensation of 3000 JPY (approximately 30 USD) for a participant of fMRI or 1000 JPY (approximately 10 USD) for a participant of human opponent.

Stimuli were presented on an MRI-compatible 32 inch LCD display with a resolution of 1920 pixels by 1080 pixels (NordicNeuroLab, Norway) placed at back of the MR bore. An MRI-compatible response pad (Current Designs, USA) was used to record responses. Psychtoolbox (Brainard, 1997) running on MATLAB (Mathworks Inc.) was used to present stimuli and record the responses.

### Details of the opponents

The participants played the game against three types of opponents. One was a human opponent (HUM) who was also a student of the University of Tokyo, with the same gender as the participant. The human opponent played the game outside the scanner. The computer opponent FIX made choices according to the probability dictated by the mixed strategy of the Nash equilibrium. In the example payoff matrix shown in Figure 1B, FIX chooses the star with the probability of one-fourth and the diamond with the probability of three-fourths. The corresponding mixed strategy for the participant against FIX would be a probabilistic choice of one-third for the star and two-thirds for the diamond. The choice probability of FIX was fixed at the equilibrium rate regardless of the participant’s choices. The other computer opponent LRN learned and predicted the participant’s choices using a machine-learning algorithm (specifically, a perceptron with a sigmoid activation function: Bishop, 2006). LRN considered the choices of the participant (high/low), its own choices (high/low) and the results (win/loss) of the most recent six trials, plus the average bias of the participant toward the high choice (which was represented as a single constant term in the learning algorithm). Thus, the perceptron received 19 inputs (= 3 × 6 + 1) in each trial (represented by ***x*** below; see Supplementary TableS1 for details about each variable). The following equation was used to determine the probability of high choice for LRN:

$$ f(\boldsymbol{x})=1/\left(1+\exp \left(\boldsymbol{wx}^{\prime}\right)\right). $$

Each of the 19 elements, *w*_i_, in the weight vector ***w*** that corresponds to each of the 19 inputs (Supplementary Table S1) was updated every trial using the following equation:

$$ \Delta{w}_i=-a\frac{\partial E}{\partial{w}_i}, $$

where

$$ E={\left(T-f(\boldsymbol{x})\right)}^2. $$

The learning rate, *a*, was set to 0.25 throughout the experiment, which was determined by pilot tests. The choice data in the practice session was used to determine the initial ***w***. *T* was set equal to 1 if the participant’s choice was low choice, otherwise 0.

### Model-based analysis

We compared the following three models to explain the choice behavior of participants. The first was a standard reinforcement learning (RL) model, and the second was a winning rate maximization (WRM) model, which is a variant of an RL model. The third was a quantal response equilibrium (QRE) model that can yield an equilibrium with bounded rationality different from the Nash equilibrium (McKelvey and Palfrey, 1995; McKelvey and Palfrey, 1998). The RL model assumes that the choice probabilities are adjusted to maximize accumulated payoff outcomes for the participant. The WRM model posits that the choice probabilities are updated to maximize the participant’s winning rate. The QRE model assumes that the choice probabilities are calculated based on the expected values estimated from the opponent’s choice probability and payoff.

Both RL and WRM models assume that the participant learns the subjective values of chosen options based on the prediction error, i.e. the difference between the participant’s subjective or predicted value for the chosen option and the actual outcome. The subjective values of the high and low choices in the RL model, ${V}_{\mathrm{RL}}^{\mathrm{H}}$ and ${V}_{\mathrm{RL}}^{\mathrm{L}}$, were updated in a trial-by-trial manner according to the following equation:

$$ V{\left(t+1\right)}_{\mathrm{RL}}^{\mathrm{chosen}}=V{(t)}_{\mathrm{RL}}^{\mathrm{chosen}}+{\alpha}_{\mathrm{RL}}\left(r{(t)}_{\mathrm{RL}}-V{(t)}_{\mathrm{RL}}^{\mathrm{chosen}}\right),\mathrm{chosen}\in \left\{H,L\right\}, $$

where the parameter *r*(*t*)_RL_ indicates the reward amount that the participant received at the *t*-th trial. The parameter *α*_RL_ is the learning rate, dictating how much each update influences the learned value. Probabilities of the high and low choices were calculated using the softmax function:

$$P{(t)}_{\mathrm{RL}}^{\mathrm{H}}=\frac{1}{1+\exp \Big(-{\beta}_{RL}\Big(V{(t)}_{\mathrm{RL}}^{\mathrm{H}}-V{(t)}_{\mathrm{RL}}^{\mathrm{L}}\Big)\Big)},$$

$$P{(t)}_{\mathrm{RL}}^{\mathrm{L}}=1-P{(t)}_{\mathrm{RL}}^{\mathrm{H}},$$

where the parameter *β*_RL_ indicates the sensitivity to the learned values. When *β*_RL_ approaches zero, the choice becomes random. When *β*_RL_ increases, the choice probability of the option with larger value approaches one.

The WRM model is a variant of the RL model. The subjective values of the high and low choices in the WRM model were updated in a trial-by-trial manner according to the following equation:

$$ \mathrm{V}{\left(\mathrm{t}+1\right)}_{\mathrm{WRM}}^{\mathrm{chosen}}\!=\!\mathrm{V}{\left(\mathrm{t}\right)}_{\mathrm{WRM}}^{\mathrm{chosen}}+{\alpha}_{\mathrm{WRM}}\left(\mathrm{r}{\left(\mathrm{t}\right)}_{\mathrm{WRM}}\!-\!\mathrm{V}{\left(\mathrm{t}\right)}_{\mathrm{WRM}}^{\mathrm{chosen}}\right)\!,\\ \mathrm{chosen}\in \left\{\mathrm{H},\mathrm{L}\right\}\!, $$

where *α*_WRM_ is the learning rate. Probabilities of the high and low choices were calculated using the softmax function:

$$P{(t)}_{\mathrm{WRM}}^{\mathrm{H}}=\frac{1}{1+\exp \Big(-{\beta}_{\mathrm{WRM}}\Big(V{(t)}_{\mathrm{WRM}}^{\mathrm{H}}-V{(t)}_{\mathrm{WRM}}^{\mathrm{L}}\Big)\Big)},$$

$$P{(t)}_{\mathrm{WRM}}^{\mathrm{L}}=1-P{(t)}_{\mathrm{WRM}}^{\mathrm{H}}.$$

In the WRM model, regardless of the amount of the outcome, *r*(*t*)_WRM_ was 1 when the participant won, otherwise *r*(*t*)_WRM_ was 0. (In the analysis, we aligned *r*(*t*)_RL_ of the RL model to be 1 or one-thirds instead of 60 or 20 JPY when the participant won, and 0 when the participant lost, so that the range of *r*(*t*) for the RL model matched with that of the WRM model.)

We also examined a QRE model to see whether participants’ choice behaviors were explained by the expected values of options. The expected value could be calculated by multiplying the opponent’s choice probability and the payoff. The probabilities of high and low choices were calculated across four fMRI runs, for each opponent of each participant. The participant’s probabilities of the high and low choices were estimated using the following equation:

$$P{(t)}_{\mathrm{QRE}}^{\mathrm{H}}=\frac{1}{1+\exp \Big(-{\beta}_{\mathrm{QRE}}\Big(E{V}^{\mathrm{H}}-E{V}^{\mathrm{L}}\Big)\Big)},$$

$$P{(t)}_{\mathrm{QRE}}^{\mathrm{L}}=1-P{(t)}_{\mathrm{QRE}}^{\mathrm{H}},$$

where *EV^H^* and *EV^L^* indicate expected values of the high and low choices, respectively.

To individually estimate the parameters of α and β from the behavioral data, we used the Broyden–Fletcher–Goldfarb–Shanno algorithm included in the Optimization Toolbox of MATLAB. The models were compared using the Akaike information criterion (AIC) (Bishop, 2006).

### Image acquisition

A 3T Prisma scanner (Siemens Medical Systems, Erlangen, Germany) was used to acquire functional images using a 64-ch head/neck coil. Mild cushioning minimized participant head movement. Sixty-eight slices of functional images were acquired using blood oxygenation level-dependent imaging (192 mm × 192 mm × 136 mm, in-plane resolution = 96 × 96, voxel size = 2 mm × 2 mm × 2 mm, thickness = 2 mm, TR = 1.5 s, TE = 25 ms, FA = 70°) using multi-band gradient-echo echo-planar sequences (Feinberg *et al.,* 2010; Moeller *et al.,* 2010; Xu *et al.,* 2013) with multi-band factor = 4. The slices were rotated 30 degrees from the AC-PC plane to the forehead to minimize the artifact due to the sinus. The images covered the entire cerebrum after the rotation. We acquired 320 volumes in each fMRI run of the main experiment and 196 volume scans in the functional localizer task.

### Image pre-processing

We used SPM12 (Wellcome Department of Cognitive Neurology, University College London) in MATLAB to process the scanned images. We performed slice-timing correction using the middle slice as a reference, scan-to-scan realignment, normalization to the EPI template of SPM12, resampling the images with the voxel size of 2 mm × 2 mm × 2 mm and spatial smoothing (full width at half maximum of isotropic Gaussian kernel = 8 mm). A high-pass filter of 128 s was used to remove low frequency noise in the main and localizer experiments.

### Image processing

As many studies have revealed that the ventral striatum (VS) and ventromedial prefrontal cortex (VMPFC) are involved in learning behaviors based on RL (O’Doherty *et al.,* 2003; O’Doherty *et al.,* 2004; Seymour *et al.,* 2004; see also Sutton and Barto, 1998), we examined whether the VS and VMPFC were associated with the prediction error of option value Δ*V*_WRM_ in the first general linear model analysis (GLM1). The conditions of opponent type (HUM/FIX/LRN) were modeled for both the choice phase and feedback phase. GLM1 included the parametric modulations of prediction error Δ*V*_WRM_ in the feedback phase for the opponent types separately.

We also included the brain activation for the high choice and low choice in each of the conditions analyzed in the second general linear model analysis (GLM2). For the choice phase, we prepared six conditions by combining choice (high/low) × opponent (HUM/FIX/LRN). The duration for each trial was measured from the onset (appearance of the choice options) to the button press indicating a choice. For the feedback phase, we prepared six conditions by combining result (win/loss) × opponent (HUM/FIX/LRN). The duration of these conditions was 2 s from the onset (appearance of feedback). The condition for button presses was also included in the design matrix.

### Region of interest analysis for RTPJ and LTPJ

To analyze the activation in RTPJ and LTPJ, we performed an analysis of regions of interest (ROIs) (Poldrack, 2007). The RTPJ and LTPJ were individually identified using a functional localizer task for ToM that was performed after the main task (Dodell-Feder *et al.,* 2011). In the localizer task, participants were first presented with stories about human agents who held false beliefs (false belief condition: Figure S1, upper panel) or stories about outdated physical objects (false photo condition: Figure S1, lower panel); after each, participants were queried for their inferences about the respective situations.

For the ROI definition, the peak coordinates of RTPJ and LTPJ were first identified in the group-level analysis, using the contrast of false belief condition *vs* false photo condition. Then, the individual peak within 10 mm from the group peak was identified in RTPJ and LTPJ. The ROI of RTPJ/LTPJ was individually defined as an 8 mm sphere centered at the individual peak. Not all individual ROIs overlapped due to their sizes and positions. The beta estimates in RTPJ and LTPJ in GLM2 reflecting brain activities in the choice phase and the feedback phase were extracted using MarsBaR (Brett *et al.,* 2002) and sent to further statistical analyses.

We also performed a multivariate pattern analysis to examine the similarity of brain activation patterns during the game against the three types of opponents. Here, we focused on the brain activity in the ROI during the choice phase. Because we were concerned with the overall similarity (or distance) of the LTPJ/RTPJ activation patterns in the HUM condition with those in the FIX and LRN conditions, we used representational similarity analysis (RSA, Diedrichsen *et al.,* 2018), rather than pattern classification analysis (e.g. support vector machine, which evaluates the accuracy of classification of an activation pattern in an ROI by defining a hyperplane as the border between the conditions: Haxby *et al.,* 2014). We first calculated spatial correlations (Spearman’s rank-order correlation) between the beta estimates for the six conditions of choice (high/low) × opponent (HUM/FIX/LRN) and then created a representational distance matrix (RDM, Nili *et al.,* 2014; Diedrichsen and Kriegeskorte, 2017) by subtracting these correlations from one. The RDM in this study was a symmetric 6 × 6 matrix, where the off-diagonal elements indicated the distances of pairs among the six (choice × opponent) conditions.

To see which artificial agent (FIX or LRN) solicited participant responses that better represented their response patterns to actual human opponents (HUM) at the neural level (i.e. activities of LTPJ/RTPJ identified by the functional localizer with the ToM task), we first collapsed high and low choices and then compared the Fisher-*z*-transformed Spearman’s correlation of HUM–LRN with that of HUM–FIX. We hypothesized that the activity pattern for HUM would be significantly more similar to that for LRN (the learning agent) than to FIX (the agent using fixed mixed strategy), because LRN (like HUM) was responsive to the participants’ choices in a bilateral manner, while FIX was not.

## Results

### Behavioral results

**Fig. 2:**
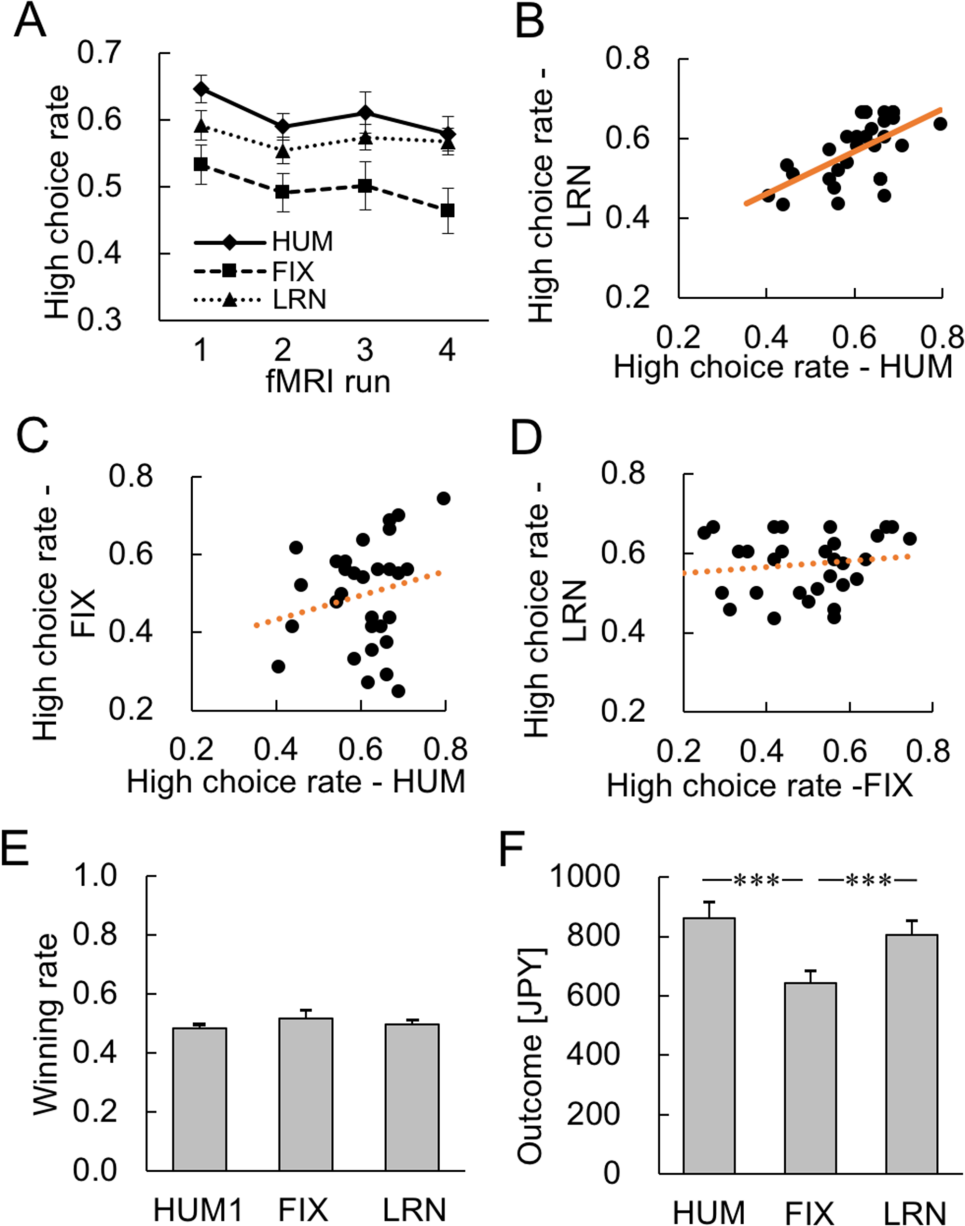
Behavioral results. (A) Percentage of high choices in four fMRI runs. The participants significantly reduced their high choice rates over time. A planned contrast between HUM-LRN combined and FIX was also significant. (B) Significant correlation of high choice rates between HUM and LRN conditions (*r* = 0.60, *P* < 0.001). (C) No significant correlation of High choice rates between HUM and FIX conditions (*r* = 0.20, *P* = 0.28). (D) No significant correlation of high choice rates between FIX and LRN conditions (*r* = 0.13, *P* = 0.49). (E) No significant difference in winning rates among the opponent types. (F) Monetary outcomes in each opponent condition. The outcomes in HUM and LRN conditions (not distinguishable from each other) were significantly higher than that in FIX condition. The asterisks indicate statistical significance (^***^P < 0.001).

As shown in Fig. 2A, the participants’ high choice rates decreased over the course of the session. A two-way repeated-measures analysis of variance (ANOVA) over opponent (HUM/FIX/LRN) × fMRI runs yielded a significant main effect of fMRI run, *F*_3,87_ = 12.0, *P* < 0.001. A planned contrast between HUM–LRN combined *vs* FIX was also significant (*F*_1,29_ = 6.08, *P* = 0.020), supporting our hypothesis that participants’ choice behaviors would be similar between HUM and LRN but distinct from FIX. The percentage of high choices in the HUM condition was also significantly correlated with that of LRN condition (*r* = 0.60, *P* < 0.001; Figure 2B) but not with that of FIX condition (*r* = 0.20, *P* = 0.28; Figure 2C). The high choice rates in FIX and LRN conditions were also not correlated with each other (*r* = 0.13, *P* = 0.49, Figure 2D).

The winning rates (collapsed over the high and low choices) were all approximately 0.5 (Figure 2E; one-sample *t* test: HUM, *t*_29_ = −1.08, *P* = 0.29; FIX, *t*_29_ = 0.15, *P* = 0.88; LRN, *t*_29_ = 0.22, *P* = 0.83) and not significantly different among the three opponent conditions (one-way repeated-measures ANOVA, *F*_2,58_ = 0.41, *P* = 0.67). The monetary outcomes that participants obtained in the HUM and LRN conditions were not distinguishable from each other (Figure 2F: *t*_29_ = 0.56, *P* = 0.58), but both were significantly higher than those in the FIX condition (*vs* HUM, *t*_29_ = 5.56, *P* < 0.001; *vs* LRN, *t*_29_ = 5.83, *P* < 0.001; see also Figure S2 for details).

We confirmed that the estimated learning rate of human opponent was similar to the learning rate of LRN (which had been set to 0.25), although the WRM and RL models were different from the learning model of LRN. The learning rates estimated using the WRM and RL models for the human opponent were 0.26 ± 0.06 and 0.22 ± 0.07 (mean ± SEM), respectively, which were not significantly different from that of LRN (RL: *t*_29_ = −0.38, *P* = 0.71; WRM: *t*_29_ = 0.18, *P* = 0.86). Next, we examined the learning rate of the participant using the WRM model. The learning rates estimated in the HUM condition (0.34 ± 0.08) and the LRN condition (0.40 ± 0.06) were indistinguishable from each other (*t*_29_ = −0.80, *P* = 0.43) and correlated (Spearman’s *r* = 0.51, *P* = 0.0044 < 0.01). On the other hand, neither the correlation of the learning rates between the HUM and FIX conditions (Spearman’s *r* = −0.082, *P* = 0.67) nor that between the LRN and FIX conditions (Spearman’s *r* = 0.198, *P* = 0.29) was significant. These results indicate that learning processes were similar between the HUM condition and the LRN condition but different from the FIX condition.

The response times for choices were similar among all three opponent conditions (HUM: 511 ± 25 ms; FIX: 487 ± 22 ms; LRN: 476 ms ± 22 ms; mean ± SEM, *F*_2,58_ = 1.30, *P* = 0.28 by a one-way repeated-measures ANOVA).

### Model comparison

We compared the WRM model, RL model and QRE model in terms of goodness of fit to the participants’ behavioral choices. The AIC of the WRM model was smaller than those of the RL model and the QRE model in all three conditions (Table 1), indicating that the WRM model achieved the best fit to the participants’ choices. The posterior predictive check on the WRM model showed significantly higher match rates (relative to the chance level) in all three conditions, whereas the RL model showed a significantly higher match rate only in the FIX condition (Table S2). Furthermore, the WRM model also best explained the choice behaviors of the human opponents who played the game outside the scanner. However, it should be noted that even the best-fitting WRM model was able to capture only 58.5% of the participants’ actual choices at most (Table S2) and the difference in AIC between the WRM and RL models was also very small (Table 1). Therefore, the following analysis of imaging data used both the WRM and the RL models.

**Table 1:** AICs of the three models considered in this study

Condition	WRM model	RL model	QRE model
HUM	65.72	66.08	66.68
FIX	63.58	65.60	66.86
LRN	62.36	62.76	66.92
HUM_opponent_	66.26	67.36	67.42

Note. The row for HUM_opponent_ shows AICs of the three models when fitted to the choices of the human opponents who played the game from outside the scanner.

### Whole brain activation for ΔV_WRM_ and ΔV_RL_

**Fig. 3:**
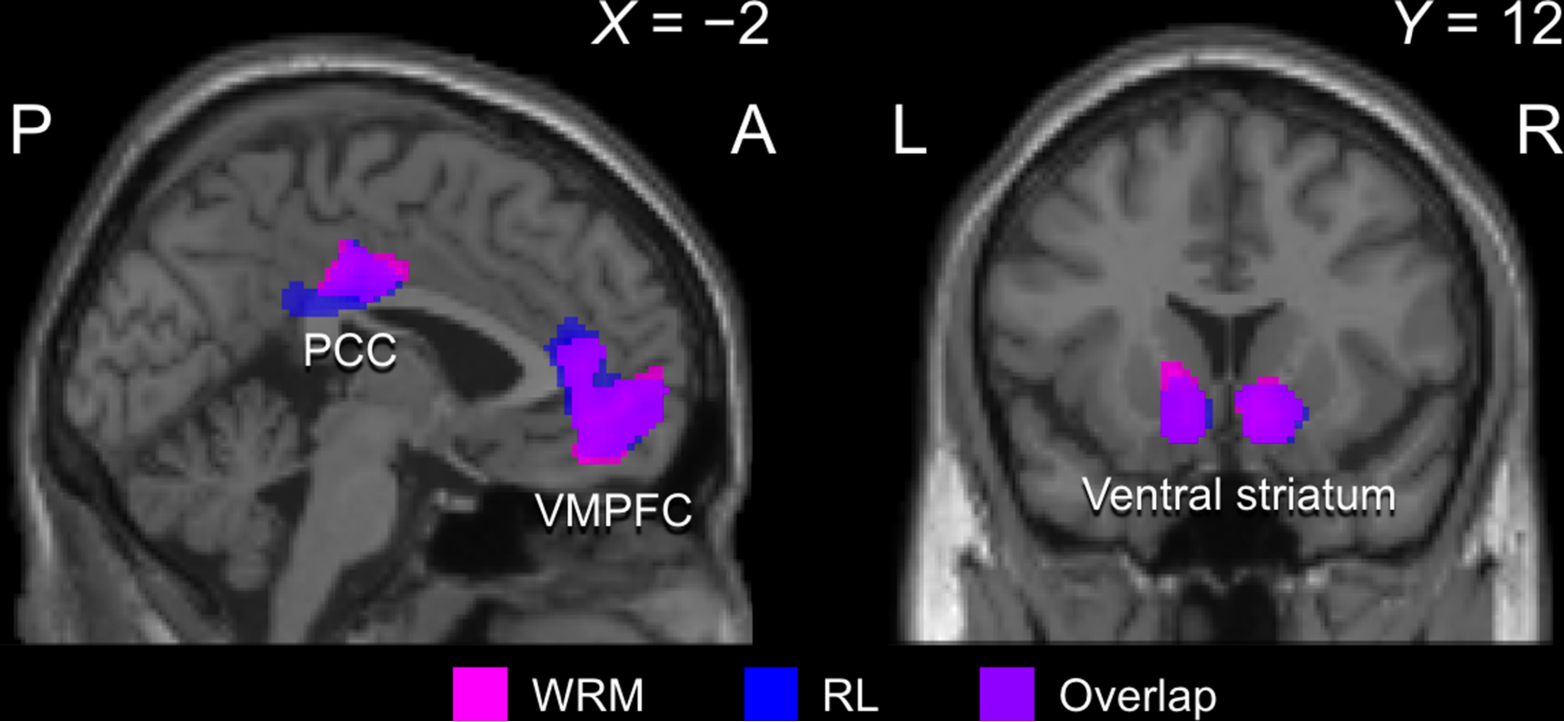
Imaging results of the whole brain analysis for the parametric modulation of prediction errors, Δ*V*_WRM_ and Δ*V*_RL_. The activation for prediction errors was observed in the VS and the VMPFC. The region colored with magenta shows the sheer activation for WRM, while the region colored with blue shows the sheer activation for RL. The overlapping region is colored violet.

The analysis for parametric modulation of Δ*V*_WRM_ and Δ*V*_RL_ in GLM1 using the standard threshold (*P* < 0.001 for cluster identification without correction, *P* < 0.05 for cluster level significance with FWE correction) showed the activation of various and large regions (Vickery *et al.,* 2011), so we used a stricter threshold (*P* < 0.05 for voxel level significance with FWE correction, cluster size threshold *k* > 50). Figure 3 shows clear activations of VS and VMPFC in response to both Δ*V*_WRM_ and Δ*V*_RL_, as in the previous imaging studies using variants of RL models (e.g. Chase *et al.,* 2015). The activities shown by the WRM and RL models (Table 2) mostly overlapped, in accord with the results of the model comparisons (Table 1).

**Table 2:** Summary of the results of parametric modulation analysis

Parameter/regions	MNI coordinates of the peak (mm)			*z* score (peak)	*P* FWE (peak)	Number of voxels
	*x*	*y*	*z*			
Δ*V_WRM_*						
VS	−14	6	−12	6.65	<0.001	482
R VS	12	12	−12	6.65	<0.001	501
VMPFC	−8	46	−10	6.26	<0.001	1379
PCC	−2	−24	38	5.82	<0.001	301
Δ*V_RL_*						
L VS	−14	6	−12	7.06	<0.001	547
R VS	12	8	−14	6.92	<0.001	567
VMPFC	0	54	−8	6.29	<0.001	1604
PCC	0	−22	36	6.46	<0.001	539

L, left; R, right; PCC, posterior cingulate cortex.

### Activation difference of RTPJ and LTPJ

**Fig. 4:**
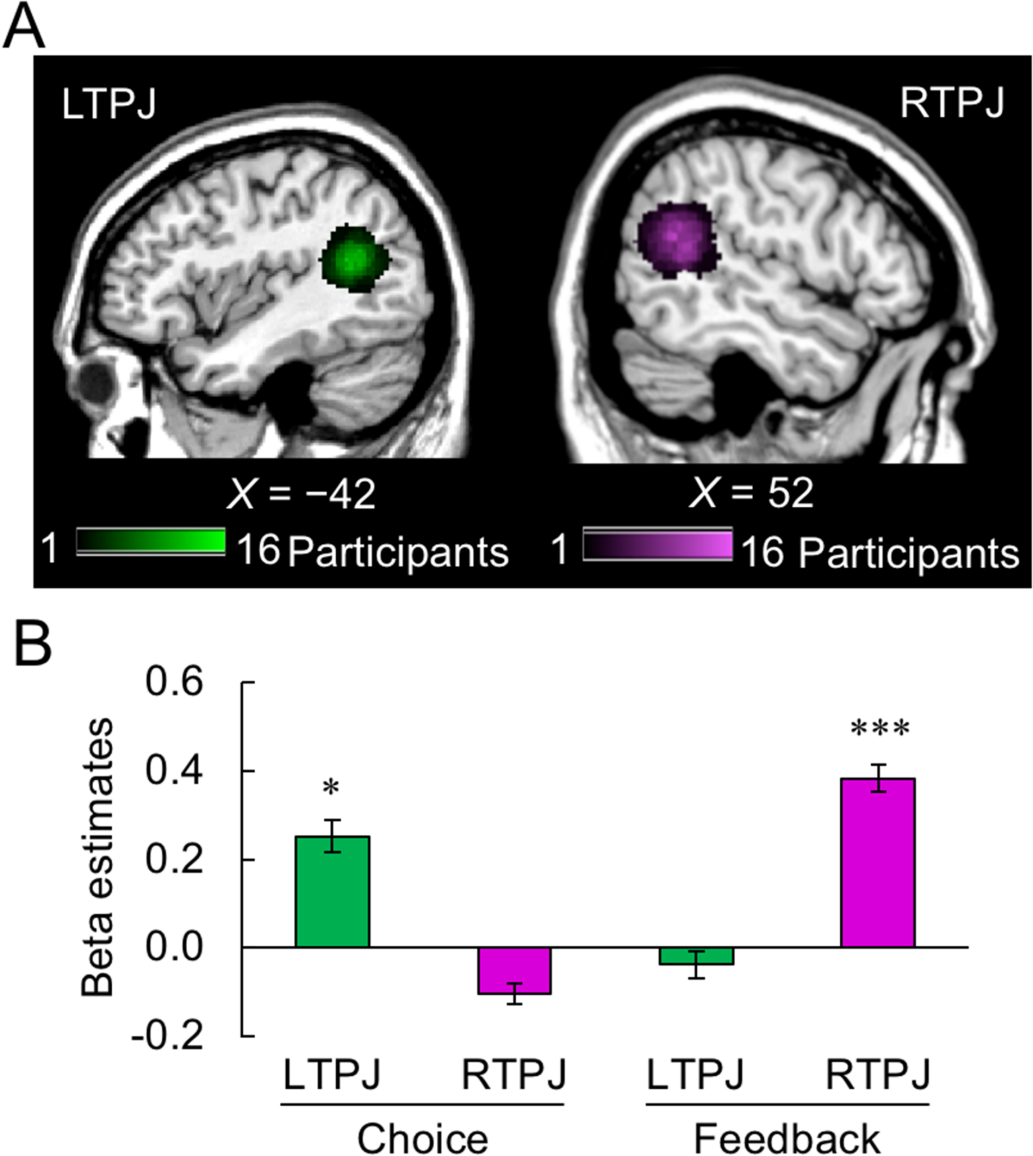
Activation in the ROIs of RTPJ and LTPJ. A. The individually defined LTPJ and RTPJ, identified using the theory-of-mind functional localizer (see Supplementary Figure S1). The color reflects the number of overlapped individual ROIs. B. Activation of LTPJ and RTPJ in the choice and feedback phases. The phase–ROI interaction was significant, indicating that the LTPJ was activated in the choice phase, while the RTPJ was activated in the feedback phase. The asterisks indicate statistical significance (^*^P < 0.05, ^***^P < 0.001).

RTPJ and LTPJ were individually identified using the functional localizer in this study (Fig. 4A, see also Methods and Figure S1). The peak coordinates in the group-level analysis with the stricter threshold (*P* < 0.05 for voxel level significance with FWE correction: Table 3) were used to identify the individual peaks in RTPJ and LTPJ and their activities while the participants played the game were extracted and compared. The LTPJ was activated in the choice phase, whereas the RTPJ was activated in the feedback phase, as shown in Figure 4B and confirmed by a significant interaction in a two-way repeated measures ANOVA [phase (choice/feedback) × ROIs (LTPJ/RTPJ); *F*_1,29_ = 24.3, *P* < 0.001]. In comparisons, the LTPJ showed the significantly higher activation than the RTPJ in the choice phase (*t*_29_ = 2.79, *P* = 0.009), and the activation of RTPJ was significantly higher than that of LTPJ in the feedback phase (*t*_29_ = 5.46, *P* < 0.001). More specifically, the activation of RTPJ was significantly higher than zero in the feedback phase (*t*_29_ = 7.31, *P* < 0.001, Bonferroni corrected for the number of ROIs) but not in the choice phase (*t*_29_ = −0.95, *P* = 0.70, Bonferroni corrected). In contrast, the activation of LTPJ was significantly higher than zero in the choice phase (*t*_29_ = 2.49, *P* = 0.019, Bonferroni corrected) but not in the feedback phase (*t*_29_ = −0.68, *P* = 0.50, Bonferroni corrected).

**Table 3:** Summary of the group-level results of the functional localizer

Contrast/regions	MNI coordinates of the peak (mm)			*z* score (peak)	*P* FWE (peak)	Number of voxels
	*x*	*y*	*z*			
False belief *vs* false photo						
RTPJ	52	−52	18	6.36	<0.001	878
LTPJ	−42	−54	18	6.72	<0.001	718

L = left, R = right.

### RTPJ activity

**Fig. 5:**
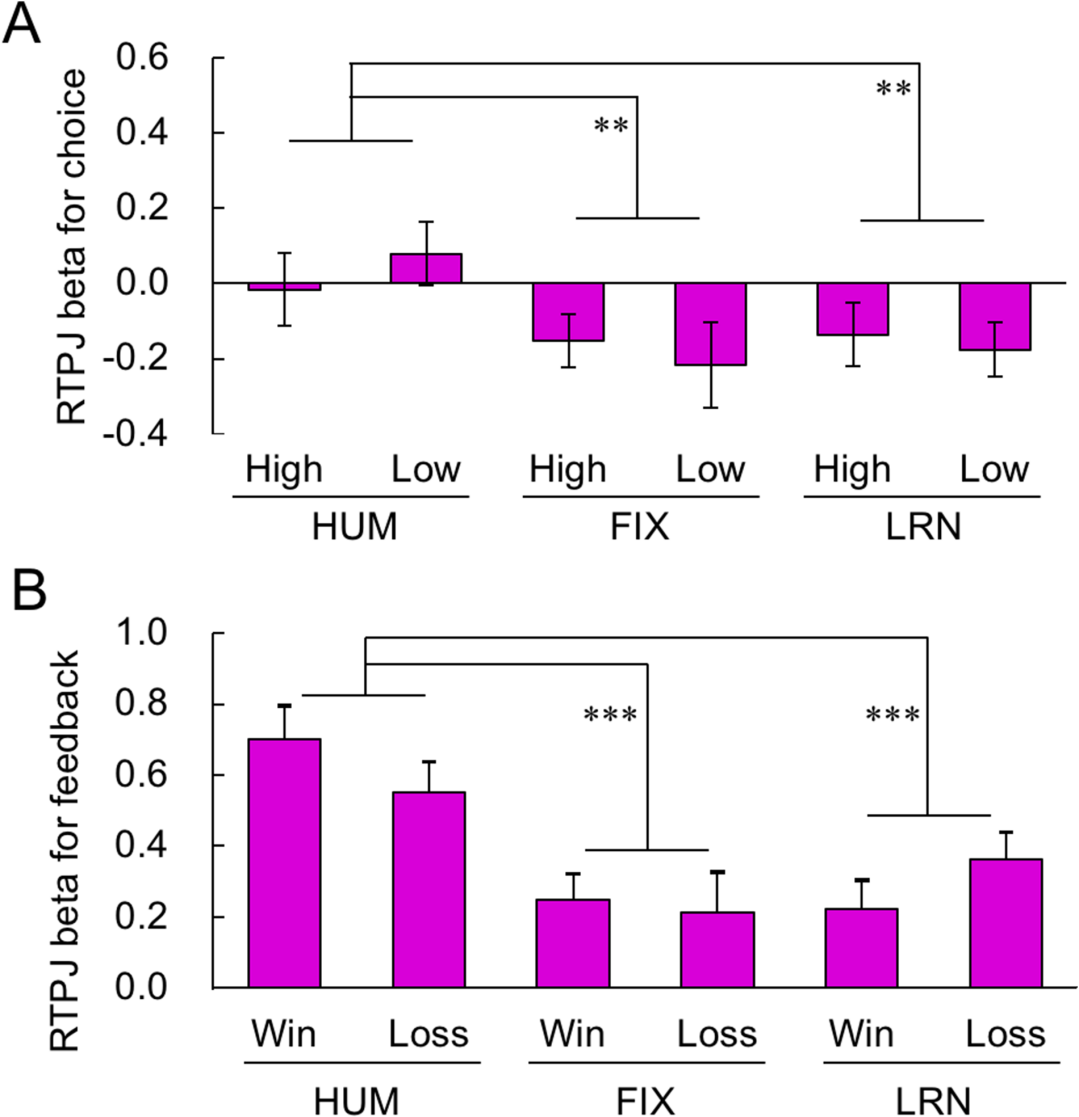
Imaging results for RTPJ. A. The activation of RTPJ in the choice phase. Although the overall activation was not statistically distinguishable from zero (see Figure 4B), the main effect of opponent was significant by a two-way repeated measures ANOVA over opponent (HUM/FIX/LRN) × choice (high/low): RTPJ activation was significantly higher in HUM than in FIX and LRN conditions. B. Activation of RTPJ in the feedback phase. RTPJ activation was significantly higher in the HUM condition than in the FIX and LRN conditions. The asterisks indicate statistical significance (^**^P < 0.01, ^***^P < 0.001).

We compared the brain activation of RTPJ in the choice phase for the combination of three opponents (HUM/FIX/LRN) and two choice options (high/low). Although the overall activation level was not statistically distinguishable from zero during the choice phase (Figure 4B left), the main effect of opponent was significant by a two-way repeated measures ANOVA (*F*_2,58_ = 6.90, *P* = 0.002 after Bonferroni correction for the number of ROIs: Figure 5A). The RTPJ activation was significantly higher in HUM than in FIX and LRN conditions (post-hoc Tukey–Kramer test, both *P* < 0.01).

As seen in Figure 5B, this main effect was also clearly observed in the feedback phase in which the overall activation of RTPJ was significantly greater than zero (Figure 4B right). A two-way repeated measures ANOVA of opponent (HUM/FIX/LRN) × result (win/loss) revealed the significant main effect of opponent type (*F*_2,58_ = 18.4, *P* < 0.001 after Bonferroni correction for the number of ROIs), and the post-hoc Tukey–Kramer test again confirmed that the brain activation in HUM condition was significantly larger than those in FIX and LRN condition (both *P* < 0.001). Taken together, these patterns suggest that the activation of RTPJ reflected the perception of human agency when the participants played against the human opponent outside of the scanner.

In line with the absence of overall activation in the choice phase (Figure 4B left), RTPJ activity had no significant relation with behavioral choices in any of the three conditions. As shown in Figure S3, correlations between participant’s high choice rate and RTPJ beta for high choice were all non-significant (HUM: *r* = 0.16, *P* = 0.39; FIX: *r* = −0.09, *P* = 0.64; LRN: *r* = 0.33, *P* = 0.08).

### LTPJ activity

We examined the LTPJ activation similarly to that of the RTPJ. LTPJ activation showed no significant effect in a two-way repeated-measures ANOVA over opponent (HUM/FIX/LRN) × choice (high/low) either in the choice phase (Figure S4A) or the feedback phase (Figure S4B).

**Fig. 6:**
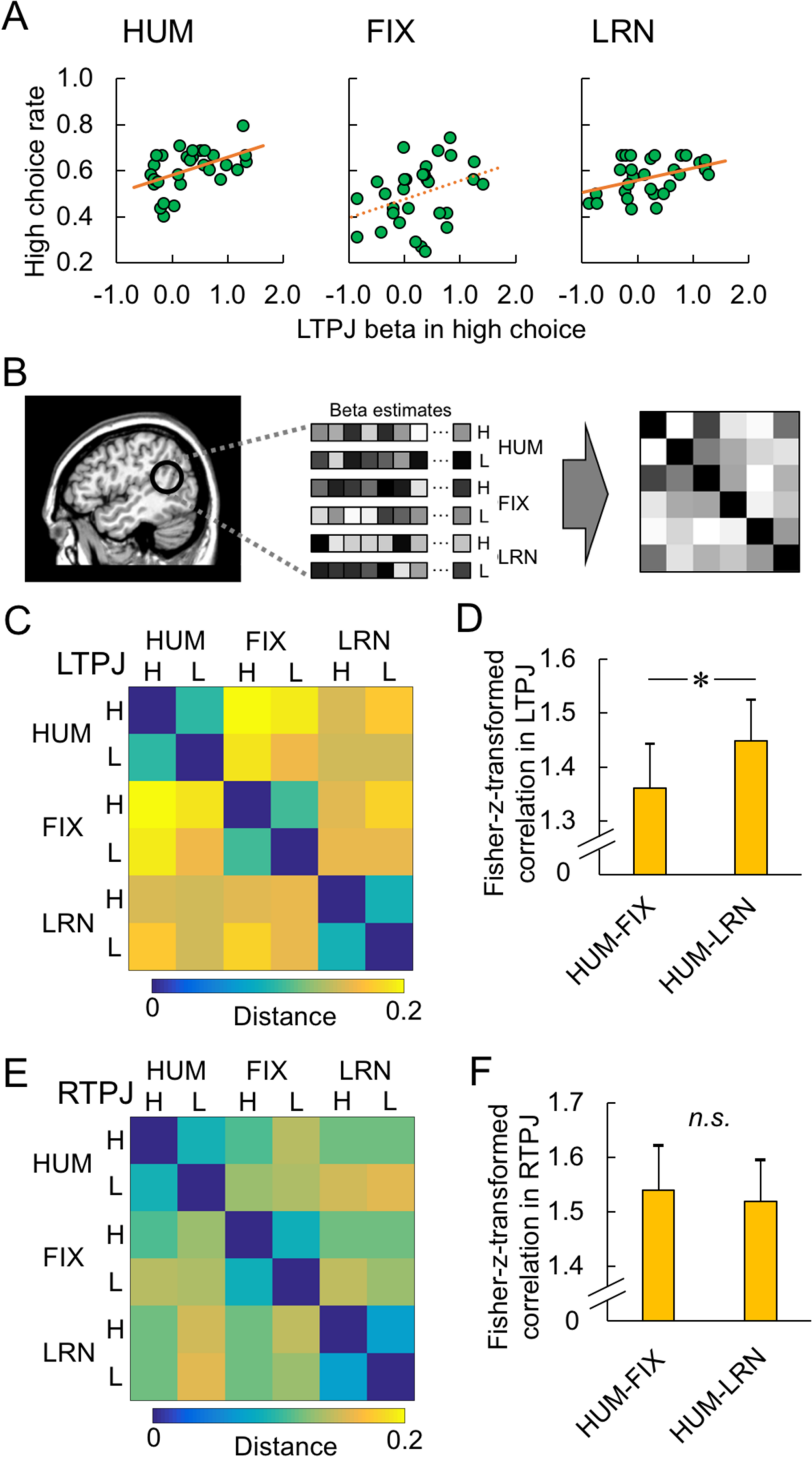
Imaging results in the LTPJ and results of RSA in the TPJs. A. The relation between LTPJ activation for high choice and the high choice rate. Significant correlation was observed in HUM and LRN conditions but not in FIX condition. B. Schema to calculate RDM. Beta estimates were extracted from the individually defined TPJ, and then the correlation matrix of the beta estimates was calculated and converted to the RDM. C. The RDM of activation in the LTPJ. The color bar shows the distance between conditions. D. The difference of Fisher-*z*-transformed correlations of HUM-FIX and HUM-LRN. The Fisher-*z*-transformed correlation of HUM-LRN was significantly higher than that of HUM-FIX. The asterisk indicates statistical significance (^*^P < 0.05). E. The RDM of activation in the RTPJ. The color bar shows the distance between the conditions. F. Similar activity patterns of RTPJ between HUM-FIX and HUM-LRN comparisons. The Fisher-*z*-transformed correlation was not significantly different between HUM-FIX and HUM-LRN.

In the behavioral results (Figure 2A and B), we observed that the high choice rate in the HUM condition was remarkably similar to the LRN condition but clearly distinct from the FIX condition. A similar pattern can be observed for LTPJ activation in the choice phase (Figure 4B left). Consistent with this conjecture, LTPJ activation for high choice was significantly correlated with the high choice rate in the HUM and LRN conditions, but this relationship was weak in the FIX condition (Figure 6A; HUM: *r* = 0.48, *P* = 0.007; FIX: *r* = 0.34, *P* > 0.10; LRN: *r* = 0.43, *P* = 0.018, Bonferroni corrected for the number of ROIs).

These results indicate that LTPJ activity in the HUM condition may be more similar to that in the LRN condition than to that in the FIX condition. We compared the activity patterns of the HUM, FIX and LRN conditions using a RSA. First, the beta estimates in the choice phase between the six conditions [choice (high/low) × opponent (HUM/FIX/LRN)] were extracted from the LTPJ ROI (Figure 6B). Next, the distance in each pair of the six conditions was defined as 1 minus the spatial correlation of beta estimates. The RDM of LTPJ indicated that the activity pattern was similar within each opponent type but less similar across the opponent types (Figure 6C). Most importantly, the Fisher-*z*-transformed correlations showed that the activation pattern of LTPJ in the HUM condition was more similar to that in the LRN condition than to that in the FIX condition (Figure 6D: *t*_29_ = 2.12, *P* = 0.042, Bonferroni corrected for the number of ROIs). However, no such dissociation among the three conditions was observed in the activation pattern in RTPJ (Figure 6E and F: *t*_29_ = 0.38, *P* = 0.71, Bonferroni corrected for the number of ROIs; in contrast to the RSA approach, the classifier approach using a support vector machine did not yield significantly higher classification above the chance level for either LTPJ or RTPJ—see Figure S5). In the feedback phase, the activation patterns in LTPJ (and also RTPJ) were not dissociable among the three conditions (Figure S6). Taken together, these results may indicate that LTPJ is involved in second-order inferences (inferring another agent’s inferences about one’s own belief) when making competitive strategic choices.

## Discussion

This fMRI study investigated the behavioral and neural bases for strategic decision making when the participant must consider an opponent’s inferences about the participant’s own beliefs in a bilateral manner. As hypothesized, the participants’ choice behaviors against the human opponent (HUM) were remarkably close to those against LRN, which learned and exploited the participant’s prior choices, as compared with those against FIX, which always followed a fixed probabilistic strategy. The model-based analyses showed that the participants’ choice behaviors were better predicted by a learning model that maximized winning rate rather than monetary outcome. We also confirmed that winning was rewarding in itself, as shown by the activation of VS and VMPFC associated with the prediction error of the winning rate (Δ*V*_WRM_). Furthermore, RTPJ and LTPJ, identified by the ToM localizer, showed double dissociation of activation and activity patterns between the choice and feedback phases. The activation of RTPJ in the feedback phase was significantly larger when playing against the human opponent (HUM) than the computer opponents (FIX and LRN). In contrast, the activity patterns of LTPJ in the choice phase showed a greater similarity between HUM and LRN conditions than between HUM and FIX conditions, paralleling the significant correlation between the participants’ choice behaviors and the corresponding LTPJ activity in HUM and LRN (but not in FIX) conditions.

As mentioned earlier, we assumed two main features of the human opponent in this study: human agency and strategic bilateral inference about the participant’s choice behaviors. Obviously, neither computer agent had human agency. But similar to HUM, LRN learned and constantly adjusted to the participant’s choice behavior for possible exploitation, while FIX did not and instead followed a fixed choice probability. The double dissociation we have observed in this study seems to reflect these two features (human agency and bilateral strategic inference). In the following, we first discuss these differential activations of RTPJ and LTPJ in more detail.

RTPJ activation was larger for the human opponent than for the computer opponents (FIX and LRN) in the feedback phase, while its activity was negligible in the choice phase and also uncorrelated with the participants’ high choice rates. In previous research using behavioral games (e.g. Sanfey *et al.,* 2003), human face pictures (in contrast to computer pictures) have often been used to increase participants’ feelings of an opponent’s human agency. However, because we were concerned that such manipulation might also evoke differences in arousal or emotional states beyond the perception of human agency, we presented only a word cue to signal that the opponent in the current trial was either a student of the same university or a computer. We still observed a significant difference in RTPJ activation between the HUM condition and the two computer conditions.

In contrast, LTPJ activation in the choice phase was correlated with high choice rate in the HUM and LRN conditions, but not in the FIX condition; behaviorally, high choice rate in HUM was also correlated with that in LRN, but not with FIX. Moreover, LTPJ activity patterns, as identified by the RSA, were similar between the HUM and LRN conditions, but not between HUM and FIX. These behavioral and neuroimaging results indicate that the LTPJ is involved in strategic decision making against an opponent that can actively learn from the prior actions of the player and infer their future actions. Previous studies have suggested that the LTPJ is associated with perceiving differences between the mental states of self and other (Perner *et al.,* 2006; Schurz *et al.,* 2013; Arora *et al.,* 2015). A recent study by Engelmann *et al.* (2019)) also reported a similar result involving the LTPJ in the context of a trust game. These researchers found a significant correlation between strength of LTPJ connectivity (e.g. with posterior superior temporal sulcus) and investment behavior when participants made trust decisions with a human counterpart, but not when they made the same investment decisions in a non-social control game. These results corroborate the argument that LTPJ activity reflects elements of social decision making.

Taken together, our results showed a functional dissociation of RTPJ and LTPJ in a strategic decision making context. The RTPJ is involved in the perception of the human agency that constitutes a basis for reasoning about the opponent’s mental state (Saxe and Kanwisher, 2003; Saxe and Wexler, 2005; Saxe, 2006; Völlm *et al.,* 2006; Van Overwalle, 2009; Van Overwalle and Baetens, 2009; Schnell *et al.,* 2011; Van Overwalle, 2011; Takahashi *et al.,* 2014), whereas the LTPJ may be involved in strategic planning of choices against intelligent, bilaterally-responsive agents, be they human or non-human.

In this study, we identified the ROIs of LTPJ and RTPJ for each of the participants separately, using the ToM localizer (Dodell-Feder *et al.,* 2011). Notice that the individual identification of functional brain regions was used not only for comparing activation between conditions but also for comparing activity patterns through RSA. The former compared the averaged activities in each ROI, whereas the latter examined the voxel-scale similarities/differences of activity in the ROI. That is, these two analyses reflected different aspects of neural activity. We believe that the ToM localizer, which enables using exactly the same ROI throughout the two analyses, will thus be beneficial in future research that investigates functional dissociations between RTPJ and LTPJ in strategic decision making.

It could be argued that the asymmetric payoff matrix employed in this study may have introduced the possibility of social preferences (e.g. inequity aversion, empathy, etc.) biasing participants’ computations of value when making choices. It is true that the asymmetric payoff matrix allowed participants to earn more than the human opponent if they played the mixed strategy of the Nash equilibrium, and this may have triggered reactions of empathy or advantageous inequity aversion. We thus additionally analyzed the activation of the anterior insula (AI), which has been shown to be associated with such social preferences (Sanfey *et al.,* 2003; Singer *et al.,* 2009; Gao *et al.,* 2018; but see Chang *et al.,* 2013 indicating association of AI with other functions). The result showed no significant difference in AI activation between the win and loss feedback situations where inequity aversion could be at work (Figure S7), which could imply that its influence was less evident in the competitive context of our matching-pennies game, as compared with a distributive context (Fehr and Schmidt, 1999). However, as we did not directly assess neural correlates of inequity aversion, the absence of AI activation remains only suggestive and should be treated with caution against reverse inference (Poldrack, 2006; Poldrack, 2011). Future research should address the role of social preferences in a competitive context more directly, along with possible involvement of other brain regions (e.g. TPJ: Morishima *et al.,* 2012) in inequity aversion.

Finally, our model-based behavior analysis showed that the WRM model was the best fit to the participants’ choices, although the difference between the WRM model and the (second best) RL model in fitness was small. Activation related to the learning process specified by the WRM model and the RL model was observed (and mostly overlapped) in the VS and the VMPFC, the reward regions identified by previous studies (O’Doherty *et al.,* 2003; Haruno *et al.,* 2004; Rodriguez *et al.,* 2006; Tobler *et al.,* 2006; Jocham *et al.,* 2011; Zhu *et al.,* 2012). These indicate that winning itself worked as a reward for the participants as well as the monetary payoff. It could be argued that the participants might have felt the monetary reward in this experiment to be too small and thus focused more on winning. However, the participants were clearly instructed that the accumulated payoff outcome could be substantial and would be paid as a cash bonus. Furthermore, the accumulated payoff outcomes for the high choice were significantly larger than for the low choice in HUM and LRN conditions (Figure S2), suggesting that the outcome difference between high and low choices was meaningful for the participants. Taken together, the results indicate that winning in itself, as well as the monetary outcomes, worked as a strong reward for the participants (cf. Klasen *et al.,* 2012; Kätsyri *et al.,* 2013).

This study investigated the neural basis for inferring an opponent’s inferences about one’s own beliefs in a competitive decision making context. Choice behaviors against the human opponent and LRN were systematically different from those against FIX. The RTPJ showed significantly higher activation in HUM condition than in the computer conditions in the feedback phase, while the LTPJ activity pattern showed higher similarity between HUM and LRN conditions than between HUM and FIX conditions. These results suggest that the RTPJ is mainly associated with the perception of human agency, and the LTPJ is involved in second-order inferences (those about others’ inferences about one’s own beliefs) in competitive situations.

## Funding

This work was supported by Japan Society for the Promotion of Science (JSPS) KAKENHI (JP25118004 and JP16H06324 to T.K. and JP16K16076 and 19K07807 to A.O.) and Japan Science and Technology Agency CREST (JPMJCR17A4 (17941861) to T.K.). Support from CiSHub at the University of Tokyo is also appreciated.

## Supplementary Material

scan-18-383-File011_nsz082
