# Supplementary material for: Dissociable roles of left and right temporoparietal junction in strategic competitive interaction
Source: Soc Cogn Affect Neurosci. 2019 Oct 31;14(10):1037–48. doi: 10.1093/scan/nsz082 (PMC6970153; doi:10.1093/scan/nsz082)
Supplement: scan-18-383-File011_nsz082 [file scan-18-383-file011_nsz082.docx]

**Supplementary Materials for**

**Dissociable roles of left and right temporoparietal junction in strategic competitive interaction**

Akitoshi Ogawa^1,2,3^, Tatsuya Kameda^4, 2^

*^1^Faculty of Medicine, Juntendo University, 2-1-1 Hongo, Bunkyo-ku, Tokyo 113-8421, Japan.*

*^2^Brain Science Institute, Tamagawa University, 6-1-1 Tamagawagakuen, Machida, Tokyo 194-8610.*

*^3^Labratory for Symbolic Cognitive Development, RIKEN Center for Biosystems Dynamics Research, 2-1 Hirosawa, Wako-shi, Saitama 351-0198, Japan.*

*^4^Department of Social Psychology, The University of Tokyo, 7-3-1 Hongo, Bunkyo-ku, Tokyo 113-0033, Japan.*


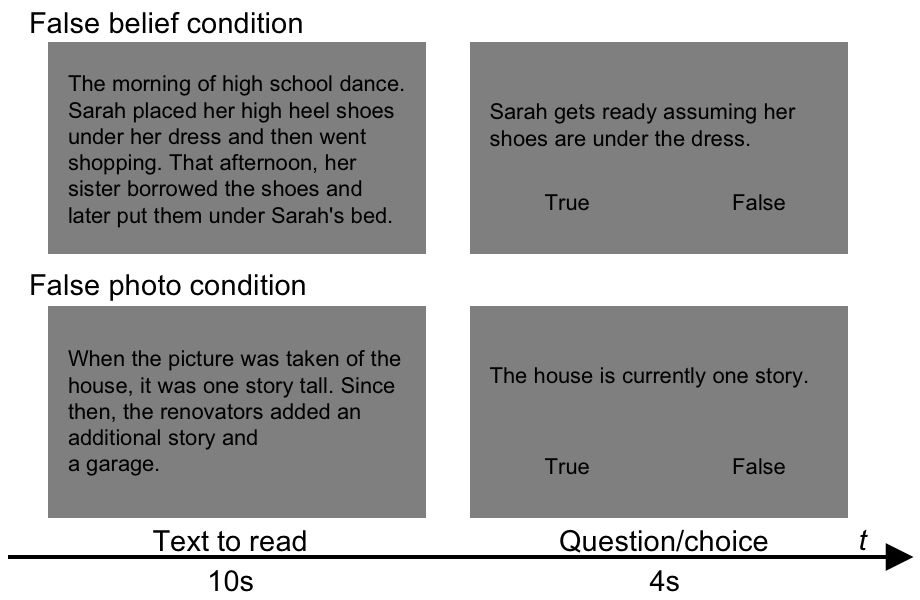


**Fig. S1.** Example trials for the functional localizer for theory of mind (Dodell-Feder et al., 2011). Participants made inferences about the false beliefs of human agents (False belief condition) or about outdated physical objects (False photo condition). There were 7 trials for each condition, with inter-trial interval of 7 s. The order of conditions was random. The contrast of these two conditions, where False belief > False photo, showed activation in the RTPJ and LTPJ for perspective taking.


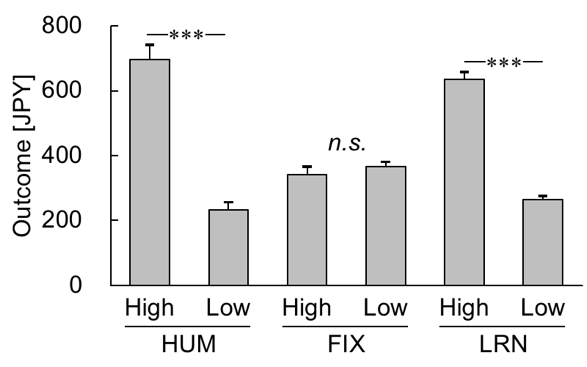


**Fig. S2.** Monetary outcomes for High and Low choices against each opponent. A two-way repeated-measures ANOVA over opponent (HUM/FIX/LRN) × choice (High/Low) revealed a significant interaction effect (*F*_2,58_ = 24.6, *P* < .001). Multiple comparisons using the Tukey-Kramer method showed that the outcome for High choices was significantly higher than that for Low choices in HUM and LRN conditions (HUM, *P* < .001; LRN, *P* < .001), but this was not the case in FIX condition (*P* = .53). Asterisks indicate statistical significance (****P* < .001).


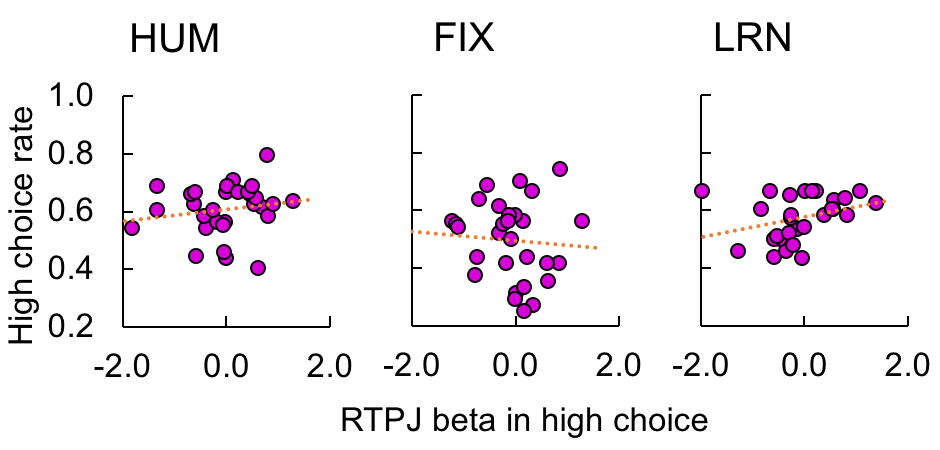


**Fig. S3.** The relation between High choice rate and RTPJ activity in the choice phase. None of the correlations in the three opponent conditions was significant (HUM, *r* = .16, *P* = .39; FIX, *r* = −.09, *P* = .64; LRN, *r* = .33, *P* = .08).


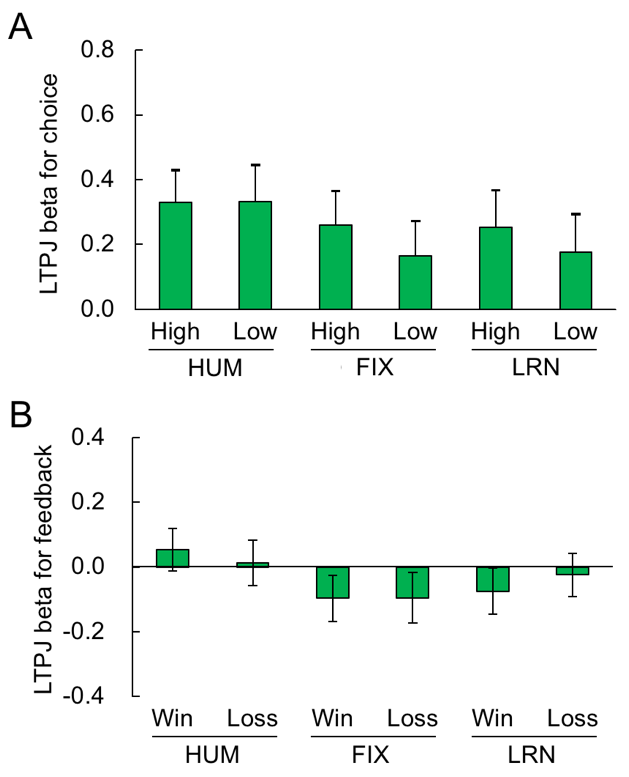


**Fig. S4.** Results of ROI analysis of LTPJ. **A.** Activity in the choice phase. No significant effect was found in a two-way repeated-measures ANOVA over opponent (HUM/FIX/LRN) × choice (High/Low), whereas the collapsed activation was significantly greater than zero. **B.** The beta estimates of feedback phase. No significant effect was found in a two-way repeated-measures ANOVA over opponents × choices, and the collapsed activation was not different from zero.


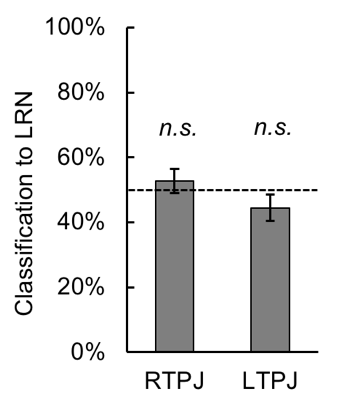


**Fig. S5.** The classification results of MVPA in the RTPJ and LTPJ using the support vector machine. In this analysis, we trained two decoders that discriminated between the experimental conditions (FIX/LRN) using the ROI activation patterns – one for LTPJ and one for RTPJ. These decoders were then fed the activation patterns in the HUM condition to test whether they could successfully classify them as belonging to LRN rather than to FIX. However, the decoders showed no significant increase in the percentage of classification to LRN above the chance level of 50% (Sign-rank test, RTPJ, *z* = 0.56, *P* = .56; LTPJ, *z* = −1.33, *P* = .18). Moreover, the decoders for LTPJ and RTPJ showed no significant difference in the percentage of classification (Sign-rank test, *z* = 1.42, *P* = .15).


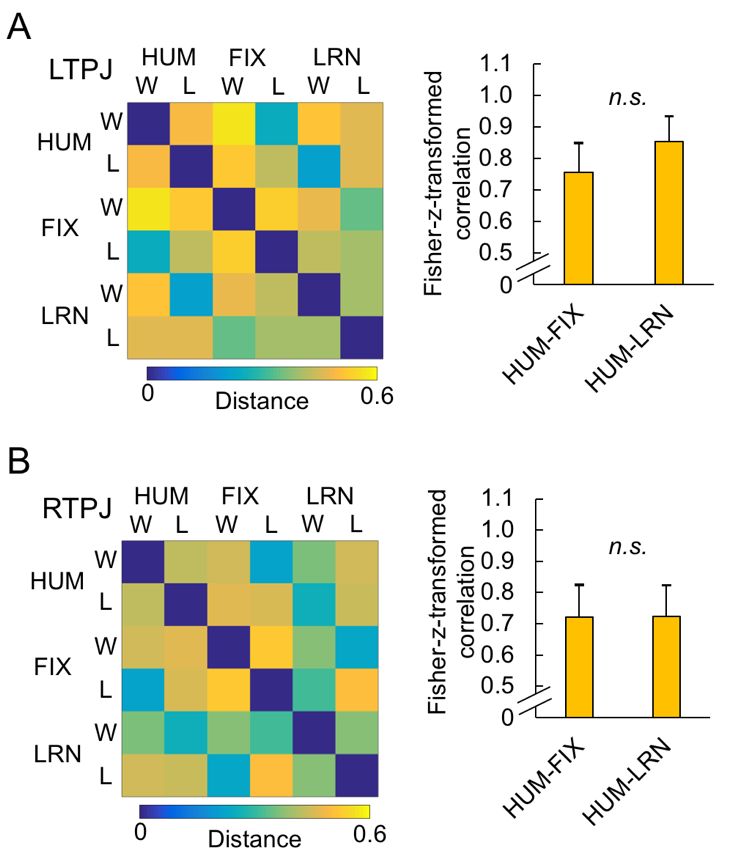


**Fig. S6.** Results of representational similarity analysis of LTPJ and RTPJ in feedback phase. **A**. Representational Distance Matrix (RDM) of activity in the LTPJ. The Fisher-z-transformed correlation was not significantly different between HUM-FIX and HUM-LRN. **B**. RDM of activity in the RTPJ. The Fisher-z-transformed correlation was not significantly different between HUM-FIX and HUM-LRN.


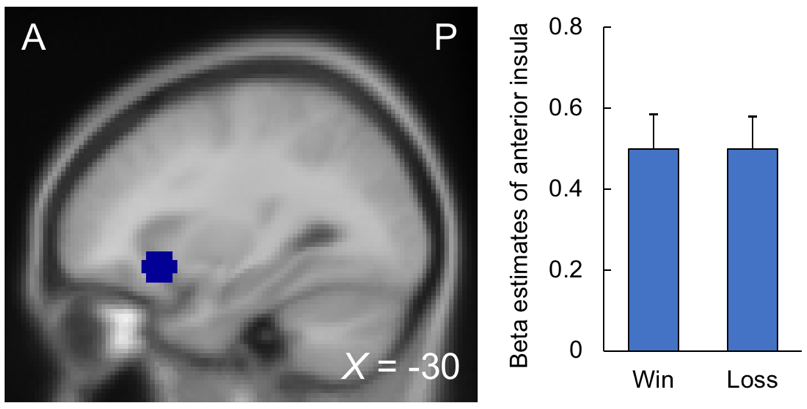


**Fig. S7.** Activation of left anterior insula in the feedback phase. We defined the left anterior insula ROI as an 8mm radius sphere centered at the previously reported peak (x = −30, y = 21, z = −20 in MNI coordinates, left panel) associated with advantageous inequity aversion (Gao *et al*., 2018). We extracted the beta estimates in the anterior insula in the Feedback phase using MarsBaR and compared the extracted values in the Win and Loss conditions. The result showed no significant difference between Win and Loss (*t*_29_ = 0.002, *P* = .998, right panel).

**Table S1.** Summary of weight and input vectors for LRN in the *t*-th trial.

| Weight *w_i_* | trial | Input *x_i_*, condition |
| --- | --- | --- |
| *w_1_* | *t* - 6 | *x_1_* =1, participant’s choice was High |
| *w_2_* | *t* - 6 | *x_2_* =1, LRN’s choice was High |
| *w_3_* | *t* - 6 | *x_3_* =1, LRN won |
| *w_4_* | *t* - 5 | *x_4_* =1, participant’s choice was High |
| *w_5_* | *t* - 5 | *x_5_* =1, LRN’s choice was High |
| *w_6_* | *t* - 5 | *x_6_* =1, LRN won |
| *w_7_* | *t* - 4 | *x_7_* =1, participant’s choice was High |
| *w_8_* | *t* - 4 | *x_8_* =1, LRN’s choice was High |
| *w_9_* | *t* - 4 | *x_9_* =1, LRN won |
| *w_10_* | *t* - 3 | *x_10_* =1, participant’s choice was High |
| *w_11_* | *t* - 3 | *x_11_* =1, LRN’s choice was High |
| *w_12_* | *t* - 3 | *x_12_* =1, LRN won |
| *w_13_* | *t* - 2 | *x_13_* =1, participant’s choice was High |
| *w_14_* | *t* - 2 | *x_14_* =1, LRN’s choice was High |
| *w_15_* | *t* - 2 | *x_15_* =1, LRN won |
| *w_16_* | *t* - 1 | *x_16_* =1, Participant’s choice was High |
| *w_17_* | *t* - 1 | *x_17_* =1, LRN’s choice was High |
| *w_18_* | *t* - 1 | *x_18_* =1, LRN won |
| *w_19_* |  | *x_19_* = 1, always |

If the condition was unsatisfied, *x_i_* = 0.

**Table S2.** Summary of the posterior predictive check of the models. The WRM model showed significantly higher match rates (relative to the chance level) in all three conditions (HUM, *t*_29_ = 18.1, *P* < .001; FIX, *t*_29_ = 3.91, *P* < .001; LRN, *t*_29_ = 3.34, *P* = .0012), whereas the RL model showed a significantly higher match rate only in the FIX condition (*t*_29_ = 4.86, *P* < .001).

| HUM  WRM | HUM  RL | FIX  WRM | FIX  RL | LRN  WRM | LRN  RL |
| --- | --- | --- | --- | --- | --- |
| 58.5% | 46.8% | 57.0% | 54.0% | 53.3% | 42.5% |
